# Anti-tumour effects of antimicrobial peptides, components of the innate immune system, against haematopoietic tumours in *Drosophila mxc* mutants

**DOI:** 10.1242/dmm.037721

**Published:** 2019-06-18

**Authors:** Mayo Araki, Massanori Kurihara, Suzuko Kinoshita, Rie Awane, Tetsuya Sato, Yasuyuki Ohkawa, Yoshihiro H. Inoue

**Affiliations:** 1 Department of Insect Biomedical Research, Centre for Advanced Insect Research Promotion, Kyoto Institute of Technology, Kyoto 606-0962, Japan; 2 Medical Institute of Bioregulation, Kyushu University, Kyushu 812-0054, Japan

**Keywords:** *Drosophila*, Innate immunity, AMPs, Lymph gland, Fat body, Cytotoxicity

## Abstract

The innate immune response is the first line of defence against microbial infections. In *Drosophila*, two major pathways of the innate immune system (the Toll- and Imd-mediated pathways) induce the synthesis of antimicrobial peptides (AMPs) within the fat body. Recently, it has been reported that certain cationic AMPs exhibit selective cytotoxicity against human cancer cells; however, little is known about their anti-tumour effects. *Drosophila mxc^mbn1^* mutants exhibit malignant hyperplasia in a larval haematopoietic organ called the lymph gland (LG). Here, using RNA-seq analysis, we found many immunoresponsive genes, including those encoding AMPs, to be upregulated in these mutants. Downregulation of these pathways by either a *Toll* or *imd* mutation enhanced the tumour phenotype of the *mxc* mutants. Conversely, ectopic expression of each of five different AMPs in the fat body significantly suppressed the LG hyperplasia phenotype in the mutants. Thus, we propose that the *Drosophila* innate immune system can suppress the progression of haematopoietic tumours by inducing AMP gene expression. Overexpression of any one of the five AMPs studied resulted in enhanced apoptosis in mutant LGs, whereas no apoptotic signals were detected in controls. We observed that two AMPs, Drosomycin and Defensin, were taken up by circulating haemocyte-like cells, which were associated with the LG regions and showed reduced cell-to-cell adhesion in the mutants. By contrast, the AMP Diptericin was directly localised at the tumour site without intermediating haemocytes. These results suggest that AMPs have a specific cytotoxic effect that enhances apoptosis exclusively in the tumour cells.

## Abstract (precis)

**Summary:** Antimicrobial peptides can interact with tumour cells generated in a haematopoietic tissue in *Drosophila mxc* mutants and have a tumour-suppressive effect on their growth.

## INTRODUCTION

Multicellular organisms have developed robust defence mechanisms to combat invading microbial pathogens that share the same environment. Insects rely fundamentally on multifaceted innate immunity, involving both humoral and cellular defence responses, as they lack an acquired immune system (Brennan and Anderson, 2004; Buchon et al., 2014; Govind, 2008; Hoffmann, 2003). The hallmark of the humoral defence mechanism is the systemic antimicrobial response. This response involves the synthesis of antimicrobial peptides (AMPs), induced by the challenged immune system, in the fat body; the fat body is a major immunoresponsive tissue that has a similar function to the mammalian liver. AMPs secreted into the haemolymph destroy invading microorganisms (Lemaitre and Hoffmann, 2007; Zhang and Gallo, 2016). Seven distinct AMPs, together with their isoforms, have been identified in *Drosophila melanogaster*: Drosomycin, Defensin, Diptericin, Metchnikowin, Attacin-A, Cecropin A2 and Drosocin (Lemaitre and Hoffmann, 2007; Martinelli and Reichhart, 2005). A series of genetic analyses have demonstrated that the genes encoding these AMPs are regulated by two major innate immune signalling pathways (Buchon et al., 2014; Hoffmann and Reichhart, 2002; Tzou et al., 2002). One of these is the Toll-mediated pathway, which is activated mainly by fungi and Gram-positive bacteria (Lemaitre et al., 1997). Infection by these microbes initiates activation of serine protease cascades, which ultimately lead to the production of the active ligand (Jang et al., 2006; Valanne et al., 2011). The active ligand binds directly to the transmembrane receptor Toll causing it to become activated (Buchon et al., 2014; Morisato and Anderson, 1994). Activated Toll transmits the signal through several proteins in the cytoplasm. At the end of the signalling cascade, the Toll-mediated signal causes degradation of Cactus. In the absence of infection, Cactus prevents the NF-κB family transcription factor Dorsal (Dl) from entering into the nucleus. Once Cactus is degraded in response to the Toll signal, Dl proteins are free to translocate into the nucleus where they can induce the expression of antimicrobial peptide genes such as *Drosomycin* (*Drs*) (Belvin and Anderson, 1996; Brennan and Anderson, 2004; Fehlbaum et al., 1994; Hoffmann, 2003). The Toll pathway also has a crucial role in the regulation of haemocyte proliferation in lymph glands (LG) and haemocyte density in haemolymph (Chiu et al., 2005; Qiu et al., 1998).

The second innate immune signalling pathway to be activated in the antimicrobial response is the Imd-mediated pathway, which responds mainly to infection by Gram-negative bacteria. The cell-wall components of the bacteria are recognised by specific transmembrane receptors (Choe et al., 2002; Gottar et al., 2002; Lim et al., 2006; Takehana et al., 2004). This recognition leads to activation of the signalling pathway and is mediated by a multiprotein complex that incorporates the Imd protein (Choe et al., 2005). Subsequently, the protein complex phosphorylates the Relish transcription factor (Rel). This phosphorylation event triggers the cleavage of Rel (Ertürk-Hasdemir et al., 2009; Stöven et al., 2000; Tanji and Ip, 2005). The N terminus of Rel drives the expression of genes encoding antimicrobial peptides, such as the Diptericin family of genes (Dpt). The Toll-mediated and Imd-mediated pathways are equivalent to the mammalian Toll-like receptor and tumour necrosis factor receptor mediated signalling pathways, respectively (Buchon et al., 2014; Hoffmann, 2003; Rawlings et al., 2004; Valanne et al., 2011). For this reason, *Drosophila* has been regarded as a powerful model organism for the investigation of innate immune signalling pathways.

Several studies have reported crosstalk between the humoral immune response and tumours in *Drosophila* (Arefin et al., 2015; Bangi et al., 2012; Xia et al., 2015). Other studies have also provided evidence that the Toll-mediated signalling pathway is activated not only by invading microbes, but also by tumour cells (Parisi et al., 2014; Pastor-Pareja et al., 2008). In *Drosophila* leukaemia models, in which there is ectopic expression of oncogenes and simultaneous depletion of tumour suppressor genes in haemocytes, the two major innate immune pathways were dysregulated (Arefin et al., 2017). However, the details of the mechanism by which the innate immune pathway is activated in organisms harbouring tumours still needs to be clarified.

**Fig. 1.**
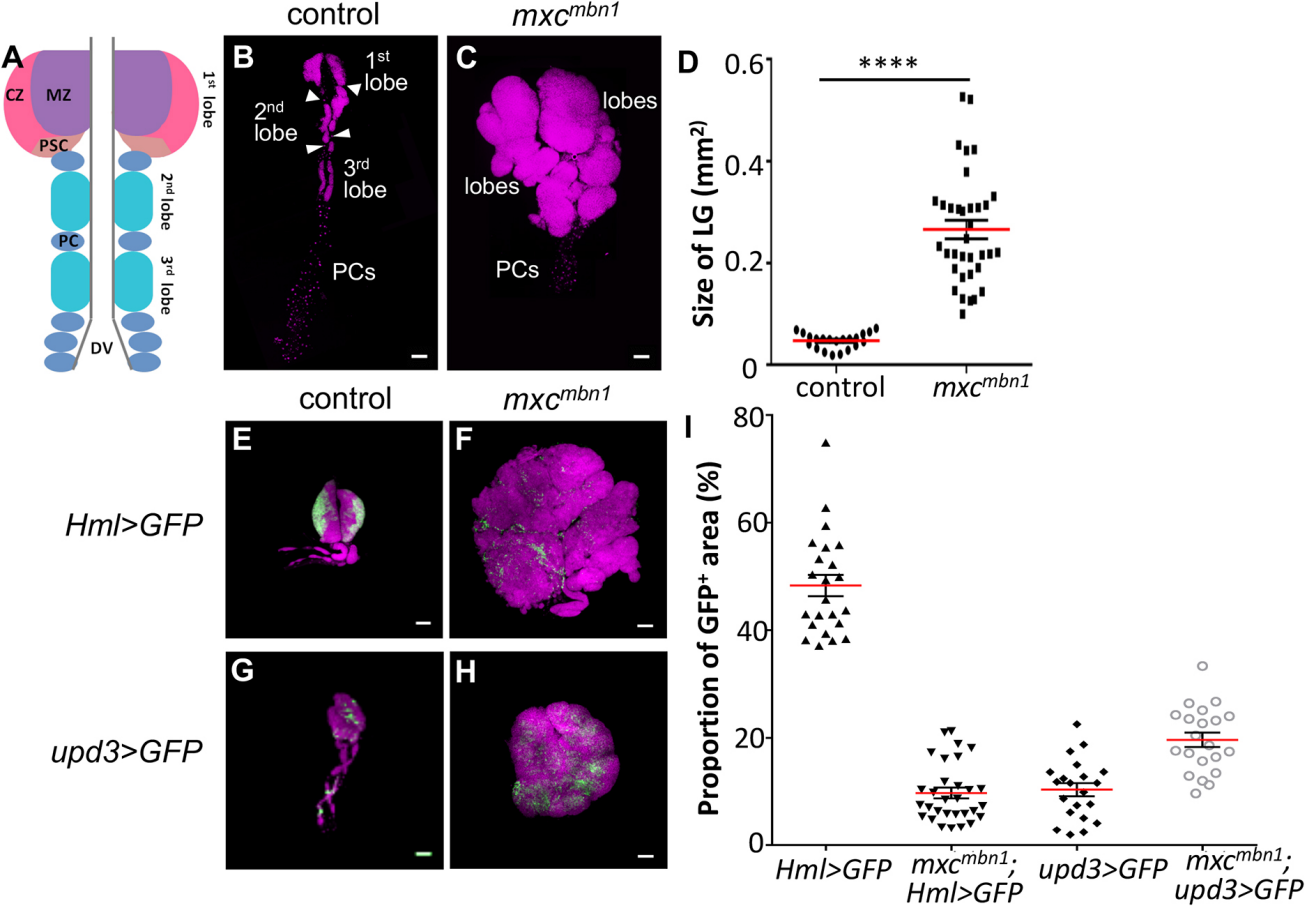
**Hyperplasia of the larval LG in *mxc^mbn1^* larvae.** (A) Schematic of the LG in *Drosophila* 3rd instar larvae. The lobes are defined as clusters of haematopoietic cells arranged in a hemispheric pattern aligned segmentally in pairs along the A-P axis of the gland and comprise a larger primary (1st) lobe, a secondary (2nd) lobe and a tertiary (3rd) lobe. The 1st lobe consists of a cortical zone (CZ), medullary zone (MZ) and posterior signalling centre (PSC). At the posterior end of these lobes, a cluster of connected pericardial cells (PCs) are lined in a row. (B) DAPI-stained whole LG isolated from normal control. Normal control LG is arranged bilaterally so that it flanks the dorsal vessel at the midline. (C) DAPI-stained whole lobes with part of the PC row isolated from hemizygous larvae (*mxc^mbn1^/Y*) for *mxc^mbn1^*. LG tumour overgrowth is visible in the *mxc^mbn1^* mutant larva. The anterior lobes are more prominently enlarged. (D) Quantification of tumour size. The right or left halves of whole LG regions of the mutant LGs (mean=0.266 mm^2^, *n*=35) are five times larger than those of normal control (mean=0.047 mm^2^, *n*=21) (Welch's *t-*test, ****P<0.0001). Error bars represent s.e.m.; red horizontal lines represent the mean. (E-H) Hyperplasia and abnormal distribution of differentiated cells labelled by *Hml>GFP* (E,F) and undifferentiated haemocyte precursors labelled by *upd3>GFP* (G,H). GFP fluorescence images of LGs prepared from normal control (E,G) and *mxc^mbn1^* mutant larvae (F,H) at mature 3rd instar stage. Magenta, DAPI staining; green, GFP fluorescence. Scale bars: 100 μm.

In addition, the cellular immune response has a significant role in protecting against invading pathogens in *Drosophila*. The circulating haemocytes that take responsibility for the cellular defence arise from two distinct haematopoietic tissues: the embryonic head mesoderm and a specialised organ designated as the LG at a later larval stage (Lanot et al., 2001; Rizki and Rizki, 1980, see Fig. 1A). The LG contains haematopoietic progenitor cells called pro-haemocytes, which can give rise to three types of haemocytes: plasmatocytes, lamellocytes and crystal cells (Evans et al., 2003; Govind, 2008; Rizki and Rizki, 1980). Some reports have shown evidence that *Drosophila* can respond to tumour cells in the body as well as to invading pathogenic microorganisms. The *Drosophila* blood cells become associated with the tumours and the innate immune system becomes activated; consequently, tumour growth is restricted (Hauling et al., 2014; Parisi et al., 2014; Pastor-Pareja et al., 2008; Wang et al., 2014). A series of studies have explored the presence of crosstalk between the cellular immune response and *Drosophila* tumours or related biological phenomena, such as a wound healing (Agaisse et al., 2003; Arefin et al., 2015, 2017; Bangi et al., 2012; Chiu et al., 2005; Kalamarz et al., 2012; Kim and Choe, 2014; Shia et al., 2009). Little is known about the mechanism by which the innate immune pathway is activated in the organisms harbouring tumours, however, or about how the activation can contribute to the suppression of tumour growth.

In this study, we focus on a blood cell tumour mutant of the *multi sex combs* (*mxc*) gene, *mxc^mbn1^*, as a *Drosophila* haematopoietic tumour model (Remillieux-Leschelle et al., 2002; Santamaria and Randsholt, 1995; Shrestha and Gateff, 1982). The *mxc^mbn1^* mutants exhibit hyperplasia in a larval haematopoietic tissue the LG, whereas they show reduced tissue growth in the imaginal discs. The mutants consequently die at the larval/pupal stage, possibly owing to the combined effects of both of these dysfunctions. Mutant larvae contain increased numbers of circulating haemocytes and abnormally differentiated haemocytes in the haemolymph. LG cells isolated from the mutant larvae could further proliferate, invade host tissues and ultimately kill the host when implanted into normal adult abdomens (Remillieux-Leschelle et al., 2002). Thus, the LG tumours in *mxc^mbn1^* can be considered as malignant tumours. The wild-type gene is also essential for the development of the imaginal discs, as larvae with mutations in some *mxc* alleles showed defective disc growth and a lack of germ cells (Landais et al., 2014; Santamaria and Randsholt, 1995; Saget et al., 1998).

To the best of our knowledge, the present study is the first to provide genetic evidence indicating that the innate immune pathways are activated in the *mxc^mbn1^* mutants carrying the LG tumours. In addition, we show that this activation is essential for the suppression of tumour progression in the haematopoietic tissue. Moreover, we demonstrate that the anti-tumour effect is exerted through the induction of AMPs, which are components of the innate immune pathways. We further observed that AMPs secreted into the haemolymph were preferentially associated with LG tumours that show reduced cell-cell adhesion, either by direct binding or with the aid of circulating haemocytes. Overexpression of the AMPs enhanced apoptosis in the LG of *mxc^mbn1^*, whereas no apoptosis occurred in control LGs. On the basis of these results, we propose that there is a surveillance system that activates the innate immune system in response to tumour cells in *Drosophila*. Moreover, AMPs might represent promising new anticancer candidates that do not have side effects.

## RESULTS

### Malignant blood neoplasm phenotype appears in larvae hemizygous for *mxc^mbn1^* mutation

Previous studies demonstrated that *mxc^mbn1^* hemizygotes showed hyperplasia of LG, a larval haematopoietic tissue, in which the precursors of haemocytes are abnormally increased in number. The LG in wild-type larva is arranged bilaterally so that the right and left halves flank the dorsal vessel at the midline (Fig. 1A). The clusters of haematopoietic cells are arranged in a hemispheric pattern on the gland and are called lobes; these are aligned segmentally in pairs along the anteroposterior axis, as a larger primary (1st) lobe, a secondary (2nd) lobe and a tertiary (3rd) lobe. At the posterior end of these lobes, a cluster of connected pericardial cells are present in a row. Each lobe in the normal LG (control) is distinctively separated by a pericardial cell (arrows and arrowheads, Fig. 1B). Whole-lobe regions of a right or left half of control LG samples prepared from a single larvae and stained with DAPI were 0.047±0.003 mm^2^ on average (*n*=21). By contrast, the LG from *mxc^mbn1^* mutants at a mature larval stage possessed enlarged lobes containing extra numbers of pre-haemocytes, thus exhibiting LG tumour overgrowth (Fig. 1C). Whole-lobe regions of the mutant LGs were 0.266±0.018 mm^2^ (data are mean±s.d.; *n*=35), five times bigger than the control (*P*<0.0001, Welch's *t-*test) (Fig. 1D). Notably, although the anterior lobes were more prominently enlarged, it was very difficult to recognise individual lobes owing to strong hypertrophy of the LGs in the mutants (Fig. 1C). The primary lobe was often undetectable or was dislocated. By contrast, the clusters of pericardial cells after anterior lobes in LGs of *mxc^mbn1^* were indistinguishable from those of controls (Fig. 1B,C). Therefore, we collected the hyperplastic anterior lobes from the mutant LGs and compared them with the corresponding regions in normal LGs.

Next, we examined which parts of the anterior lobes were excessively proliferated in the hypertrophic LGs. In the normal LG, differentiated haemocytes, which can be visualised using *Hml>GFP*, are localised at the cortical zone of the 1st lobe (Fig. 1E). By contrast, the undifferentiated haemocyte precursors, visualised using *upd3>GFP*, are enriched in the medullary zone of the 1st lobe (Fig. 1G). In the hyperplastic LGs of *mxc^mbn1^* larvae, the proportion of undifferentiated haemocyte precursors was significantly increased (*P*<0.0001, Welch's *t*-test) (Fig. 1F,I). By contrast, the proportion of undifferentiated haemocyte precursors remarkably declined (*P*<0.0001, Welch's *t*-test) (Fig. 1H,I). These observations tell us that hyperplasia of the LG mainly occurred in the medullary zone enriched in the undifferentiated haemocytes. These mutant LG cells from the larvae continue to proliferate and invade other tissues when they are injected into abdomens of normal adults (Remillieux-Leschelle et al., 2002) (K. Komatsu, M.K., Y.H.I., unpublished). Thus, it is likely that the *mxc^mbn1^* mutant exhibits a malignant phenotype in the LGs.

### RNA sequence analysis demonstrates that many immune response genes are upregulated in *mxc^mbn1^* larvae

RNA-seq analysis was carried out to identify genes with altered mRNA levels in the LG malignant hyperplasia in *mxc^mbn1^* larvae. We determined the DNA sequences of 19,529,450 cDNA reads prepared from mRNAs expressed in the wild-type mature larvae and 26,290,482 cDNA reads from *mxc^mbn1^* larvae at the same developmental stage. The RNA-seq reads were mapped to a total of 13,747 mRNAs. We found 209 genes for which the mRNA levels in the mutant were increased more than ten times compared with control mRNA levels (Table S1). By contrast, we found 320 genes for which the mRNA levels in the mutant decreased to less than 1% of control mRNA levels (Table S2). Among the downregulated genes, the peak expression for most occurred during the pupal and adult stages in the males or in the adult testes. We collected male larvae and prepared total RNAs from both controls and mutants, because the *mxc* gene is X-linked. These results are consistent with previous findings that larvae with the mutated *mxc* gene lack germline cells (Landais et al., 2014). Among the upregulated genes, 5% corresponded to tumour-related genes such as *Pvf2* and *nimC1* (K. Komatsu, M.K., Y.H.I., unpublished). Surprisingly, 31% of the upregulated genes corresponded to immunity-related genes involved in host defence against bacterial infection (Fig. S1). In this study, we focused on the upregulated immunity-related genes in the *mxc* mutant. These results led us to speculate that the *Drosophila* innate immune pathways could be activated in response to tumour cells.

### Expression of the target genes controlled by innate immune pathways is upregulated in *mxc^mbn1^* mutant larvae

**Fig. 2.**
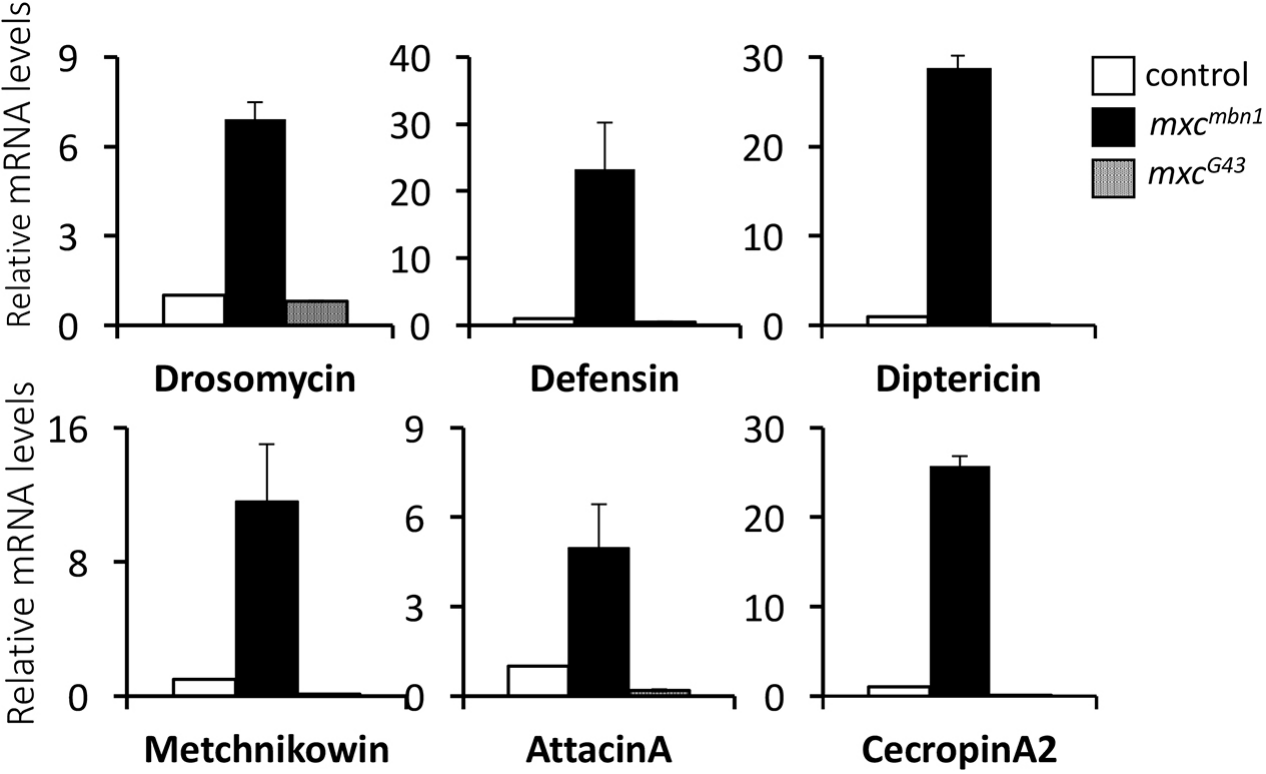
**Activation of two innate immune pathways in the *mxc^mbn1^* mutant.** The relative mRNA levels of the target genes of innate immune pathways in control (*w/Y*) larvae, tumour-harbouring larvae hemizygous for *mxc^mbn1^* and larvae hemizygous for non-tumourous *mxc^G43^*are shown as quantified by qRT-PCR. The mRNA levels for samples were normalised to control values. Error bars represent s.e.m. *Drs* and *Def* are controlled by the Toll pathway. *Dpt* is regulated by the Imd pathway. *Mtk*, *AttA* and *CecA2* are controlled by both the Toll and Imd pathways. Notably, all six AMP genes are specifically upregulated in the *mxc^mbn1^* mutant larvae.

To confirm the RNA-seq data and further investigate the genes showing altered expression, we tested whether the two major innate immune pathways (the Toll- and Imd-mediated pathways) were activated in the mutant larvae harbouring the LG tumours. First, we quantified relative mRNA levels of six antimicrobial peptide genes, *Drs*, *Defensin* (*Def*), Dpt, *Metchnikowin* (*Mek*), *Attacin A* (*AttA*) and *Cecropin A2* (*CecA2*), using quantitative real-time PCR (qRT-PCR; Fig. 2). Each of these genes is a target for one or two of the three innate immune pathways. We found that the levels of *Drs*, *Def*, *Dpt*, *Mek*, *AttA* and *CecA2* mRNAs increased in the *mxc^mbn1^* larvae by 6.9-, 23.3-, 29-, 11.6-, 5- and 26-fold, respectively, compared with those in control larvae (*w/Y*) (*P*<0.0001). We failed to detect a similar upregulation in any of the AMP genes in non-tumourous *mxc^G43^* mutant larvae, relative to the control whole larvae.

**Fig. 3.**
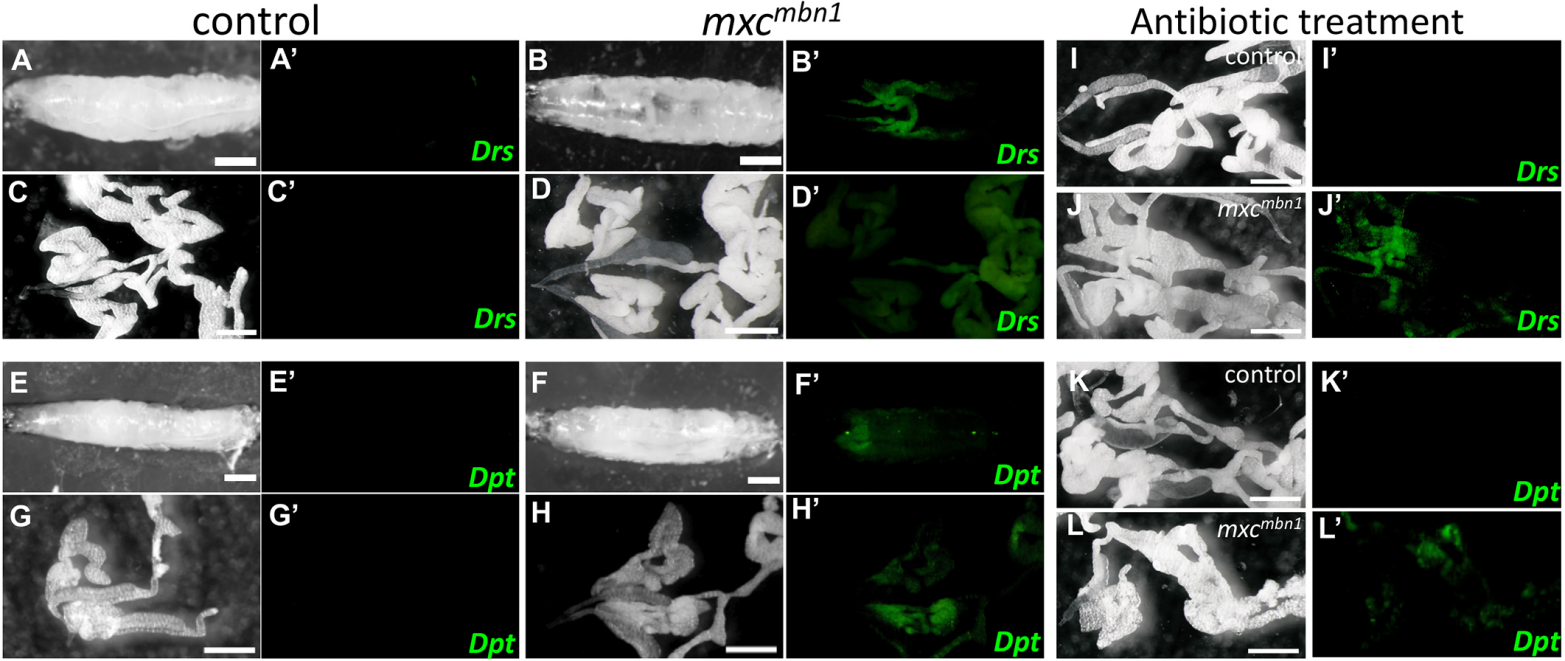
**Enhancement of AMP expression in the *mxc^mbn1^* mutant.** (A,B,E,F) Bright-field images of control (*w/Y*) (A,E) and *mxc^mbn1^* (B,F) whole larvae. (C,D,G,H,I,J,K,L) Bright-field images of the larval fat bodies around the salivary glands from the control (C,G,I,K) and *mxc^mbn1^* (D,H,J,L) larvae, raised on normal diet (A-H) or on a diet containing antibiotics (I-L). (A′,B′) Fluorescence images of whole larvae carrying *Drs-YFP* in control (A′) and *mxc^mbn1^* (B′). (C′,D′) Fluorescence images of larval fat bodies carrying *Drs-YFP* from control (C′) and *mxc^mbn1^* larvae (D′). Notably, *Drs-YFP* was induced in fat bodies of the *mxc^mbn1^* mutant, but not in the control. (E′,F′) Fluorescence images of the whole larvae carrying *Dpt-YFP* in control (E′) and *mxc^mbn1^*(F′). (G′,H′) Fluorescence images of larval fat bodies caring *Dpt-YFP* from control (G′) and *mxc^mbn1^* larvae (H′). Notably, *Dpt-YFP* was also induced in fat bodies of the *mxc^mbn1^* mutant, but not in control. (I′-L′) Fluorescence images of larval fat bodies carrying *Drs-YFP* (I′,J′) or *Dpt-YFP* (K′,L′) from control (I′,K′) and *mxc^mbn1^* larvae (J′,L′). Notably, *Drs-YFP* and *Dpt-YFP* were also induced in fat bodies of the *mxc^mbn1^* larvae raised under aseptic conditions. Scale bars: 500 μm.

Having obtained results indicating that the expression of target genes activated by two major immune pathways was significantly upregulated in the mutant larvae, we further confirmed the expression of the target gene products in the *mxc^mbn1^* larvae. Using fluorescent protein reporters that monitor *Drs* (Fig. 3A-D) and *Dpt* (Fig. 3E-H) we were able to examine activation of the Toll-mediated and Imd-mediated pathways, respectively. In the control larvae, expression of these targets was not stimulated under normal culture conditions (Fig. 3A′,C′). By contrast, expression of the *Drs* gene was substantially upregulated in the fat body, which is a key tissue in the production and secretion of AMPs, in *mxc^mbn1^* larvae (Fig. 3B′,D′). We also observed induction of *Dpt-YFP* in the fat bodies in *mxc^mbn1^* (Fig. 3F′,H′). Similarly, we detected a strong GFP fluorescence in the *mxc^mbn1^* larvae carrying *Drs-GFP* or *Dpt-YFP*, even in those raised on a diet supplemented with penicillin and streptomycin under aseptic culture conditions (Fig. 3J′,L′). These results demonstrate that the Toll-mediated and Imd-mediated pathways are activated in the fat body of the *mxc^mbn1^* larvae independent of microbial infection.

### Ectopic activation of innate immune pathways suppresses the LG phenotype of *mxc^mbn1^*

**Fig. 4.**
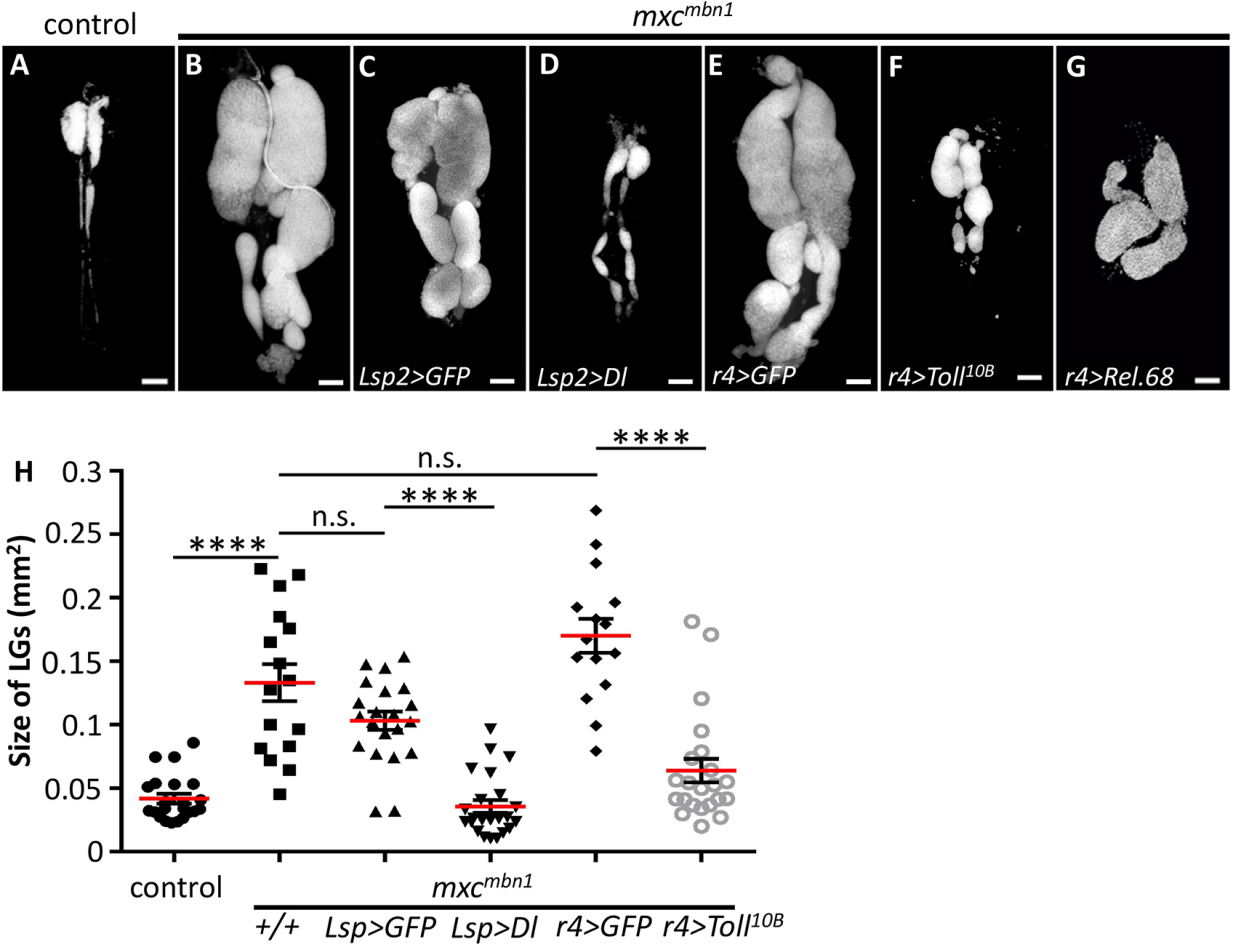
**Overexpression of innate immune pathway components suppresses LG tumour overgrowth.** (A-G) LGs stained with DAPI from mature larvae at 3rd instar stage showing a control larva (*w/Y*) (A), an *mxc^mbn1^* larva (B), LGs from *mxc^mbn1^* larvae overexpressing GFP (C) or *Dl* (D) by *Lsp2-Gal4*, or LGs from *mxc^mbn1^* larvae overexpressing GFP (E), *Tl^10B^* (constitutively active form of Toll) (F) or Rel.68 (constitutively active form of Rel) (G). (H) Quantification of tumour size (Welch's *t*-test, n.s. not significant, ****P<0.0001). Error bars represent s.e.m. The horizontal red lines represent the mean. Scale bars: 100 μm.

On the basis of the finding that the *Drosophila* innate immune pathways were activated in the *mxc^mbn1^* larvae, we tested the possibility that activation of the innate immune pathways can influence tumour development. We examined whether upregulation of these pathways could suppress the tumour overgrowth phenotype in *mxc^mbn1^* larvae. We induced ectopic overexpression of the Dl transcription factor, which participates in the Toll-mediated pathway, as well as of a constitutively active form of Toll receptor. For ectopic induction of these factors in the fat body, we used fat-body-specific Gal4 drivers. Using the drivers *ppl-gal4*, *r4-gal4* and *Lsp2-gal4*, which can induce *UAS*-dependent gene expression in fat-body cells, the amounts of *Drs* mRNA increased by 26-, 16- and 2.6-fold, respectively, compared with those in control larvae. Consequently, we observed that the ectopic activation of innate immune pathways suppressed the LG tumour overgrowth phenotype (Fig. 4). We induced the ectopic overexpression of *Dl* by a less efficient fat-body driver, *Lsp2-Gal4*, in order to avoid a higher mortality before a mature larval stage. The average size of the LG in *mxc^mbn1^/Y; Lsp2>Dl* was 34% of that in *mxc^mbn1^/Y*; *Lsp2>GFP* (*n*>20) (Fig. 4B-D,H), indicating that ectopic activation of the Toll pathway by *Dl* overexpression in fat-body cells significantly suppressed the tumour overgrowth phenotype that appeared in *mxc^mbn1^* larvae (*P*<0.0001, Welch's *t*-test) (Fig. 4C,D,H). We induced expression of a dominant allele of *Tl* or that of *Rel* by using a more efficient driver, *r4-Gal4*. The average size of the LG of *mxc^mbn1^/*Y*; r4>Tl^10B^* was 47% of that in *mxc^mbn1^/Y; r4>GFP* (*n*>20) (Fig. 4E,F), indicating that the ectopic activation of the Toll pathway by overexpression of the constitutively active form of a Toll receptor in fat-body cells significantly suppressed the tumour overgrowth phenotype (*P*<0.01, Welch's *t*-test) (Fig. 4H). Moreover, we tried to examine whether ectopic expression of the active form of the Imd pathway provides a similar suppressive effect on LG overgrowth. Consistently, we observed a distinct suppression of the LG hyperplastic phenotype in a few *mxc^mbn1^* larvae overexpressing a dominant active form of Rel (*mxc^mbn1^/Y; r4>Rel.68*) (Fig. 4E,G,H), although many of the larvae died before the mature larval stage. These observations indicate that ectopic activation of the innate immune pathway in the fat body can significantly suppress the LG tumour overgrowth phenotype in *mxc^mbn1^* larvae.

In addition, attempts were made to reduce the effect of each innate immune pathway by introducing mutations in the genes required for these pathways. By creating genetic crosses, we generated *mxc^mbn1^* larvae carrying a heterozygous amorphic mutation for individual components of the innate signalling pathways, namely *dl*, *Tl* and *imd*, and then examined whether growth of the LG tumours changed under these conditions. We noticed that the reduced gene dose of the immune factors enhanced the LG tumour overgrowth phenotype in each case (Fig. S2A-H). The average size of LGs from *mxc^mbn1^/Y; dl^4^/+* larvae was about twice that of *mxc^mbn1^/Y* (*n*>20) (*P*<0.01, Welch's *t*-test) (Fig. S2B,C,H). Also, LGs of *mxc^mbn1^/Y; Tl^rv1^/+* larvae were about twice as large as those of *mxc^mbn1^/Y* (*n*>20) (*P*<0.05, Welch's *t*-test) (Fig. S2D,H). Furthermore, the tumour overgrowth phenotype was significantly enhanced in *mxc^mbn1^* larvae carrying *Tl* mutations in both alleles, compared with that of *mxc^mbn1^* larvae heterozygous for *Tl^rv1^*(*P*<0.001, Fig. S2D,F,H). Similarly, the average size of LGs of *mxc^mbn1^/Y; imd/+* larvae was about four times that of *mxc^mbn1^/Y* (*n*>20) (*P*<0.0001, Welch's *t*-test) (Fig. S2B,E,H). The tumour overgrowth phenotype was further enhanced in *mxc^mbn1^* larvae homologous for the *imd* mutation, compared with that of *mxc^mbn1^* larvae heterozygous for *imd* (*P*<0.0001, Fig. S2E,G,H). The tumour overgrowth phenotype was enhanced in a gene dose-dependent fashion of a component in each innate immune pathway. Consistently, we showed that mRNA levels of *Drs* and *Dpt* declined remarkably in *mxc^mbn1^* larvae carrying *Tl^rv1^/Tl^r3^* and larvae homozygous for the *imd* mutation, respectively (Fig. S3), although we have not obtained clear-cut results indicating a significant reduction of the AMP mRNAs in *mxc^mbn1^* larvae heterozygous for the *Tl^rv1^* or *imd* mutation. In summary, the LG tumour overgrowth phenotype was significantly enhanced in *mxc^mbn1^* larvae carrying mutations in factors of the innate immune pathways. These genetic data are consistent with our previous results showing that ectopic activation of the Toll-mediated pathway suppressed the LG overgrowth phenotype. Taking these results together with the data mentioned previously, we conclude that the innate immune pathways can be activated in *mxc^mbn1^* harbouring LG tumour cells and, consequently, the LG tumour overgrowth phenotype can be altered.

### AMPs produced in the fat body suppress the LG tumour phenotype

**Fig. 5.**
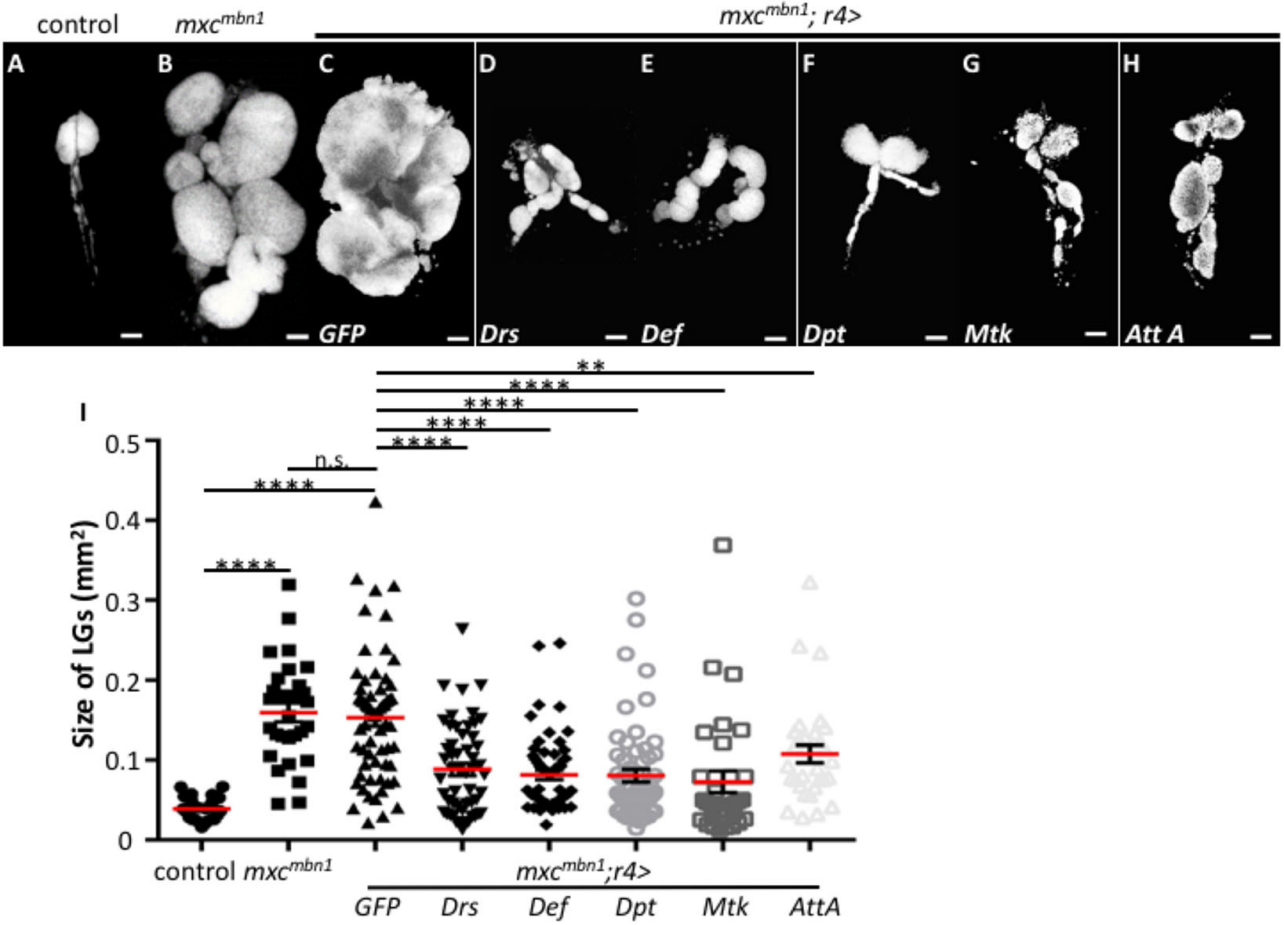
**Fat body-specific induction of AMP genes that are targets of innate immunity pathways in *mxc^mbn1^* larvae.** (A-H) LGs stained with DAPI from a control (*r4-Gal4/+*) larva (A), from *mxc^mbn1^* mutant larvae (B) or from *mxc^mbn1^* larvae expressing GFP (*mxc^mbn1^/Y; r4>GFP*) (C), *Drs* (*mxc^mbn1^/Y; r4>Drs*) (D), *Def* (*mxc^mbn1^/Y; r4>Def*) (E), *Dpt* (*mxc^mbn1^/Y; r4>Dpt*) (F), *Mtk* (*mxc^mbn1^/Y; r4>Mtk*) (G) and *AttA* (*mxc^mbn1^/Y; r4>AttA*) (H). (I) Quantification of tumour size (Welch's *t*-test, n.s. not significant, **P<0.01, ****P<0.0001). The error bars represent s.e.m. The horizontal red lines represent the mean. Scale bars: 100 μm.

The genetic data presented above allowed us to speculate that the ectopic induction of the AMP genes, which are targets of the innate immune pathways, was responsible for the suppression of the LG tumour overgrowth phenotype in *mxc^mbn1^*. To test whether the AMPs have a suppressive effect on LG tumour overgrowth, we induced ectopic expression of five different AMPs in the fat bodies of *mxc^mbn1^* larvae using the fat-body-specific *gal4* driver, *r4-gal4*. This approach revealed a significant suppression of the LG tumour overgrowth phenotype in *mxc^mbn1^* by expression of each AMP (Fig. 5B-I). The average size of the LGs in *mxc^mbn1^/Y; r4>Drs* was 58% of that in *mxc^mbn1^/Y; r4>GFP* (*n*>55), indicating that the ectopic *Drs* overexpression in the fat-body cells significantly suppressed the tumour overgrowth phenotype (*P*<0.0001, Welch's *t*-test) (Fig. 5B-D). Moreover, the average LG size in *mxc^mbn1^* mutants, expressing the other four AMPs in a similar manner, decreased to between 48% and 70% of the average size in *mxc^mbn1^* mutants without AMP overexpression (*n*>30, *P*<0.01, Welch's *t*-test) (Fig. 5I). These observations suggest that suppression of LG tumour overgrowth in *mxc^mbn1^* by activation of the two major innate immune pathways is relevant to ectopic expression of AMPs in the fat bodies. Taking into consideration the genetic results described here, we interpreted that the *Drosophila* innate immune pathways could be activated in response to the LG tumour cells and that the expression of their target genes encoding AMPs can suppress the LG tumour growth.

### Ectopic induction of AMPs in the fat body enhances apoptosis in the *mxc^mbn1^* LG, but not in normal tissues

**Fig. 6.**
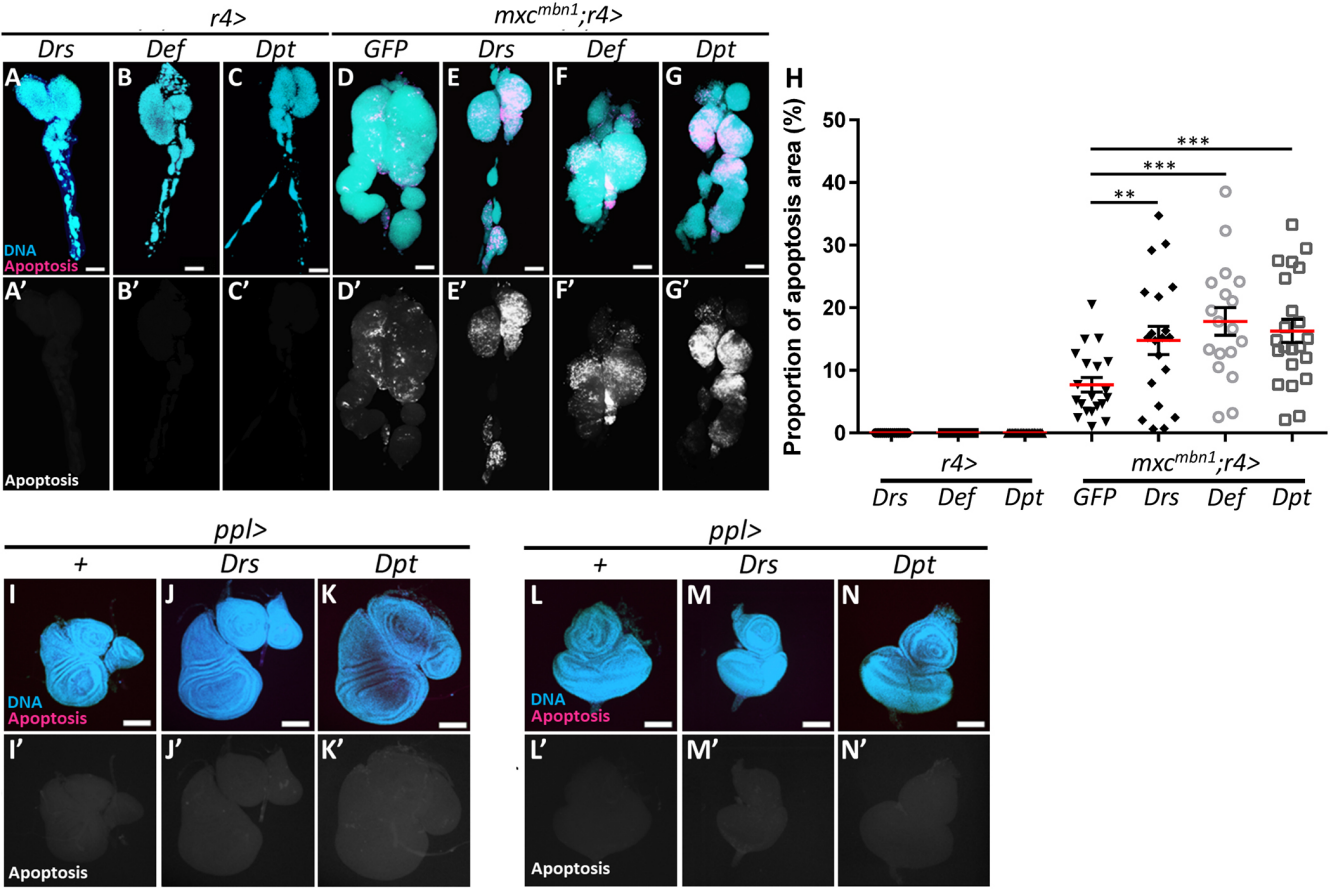
**Observation of LG cells undergoing apoptosis in *mxc^mbn1^* larvae overexpressing AMPs in** **the** **fat bodies.** (A-G,I-N) Cell Event Caspase-3/7 Detection Reagent assays in LGs (A-G), wing and haltere discs (I-K), and eye discs (L-N). Red signals in A-G and I-N, and white signals in A′-G′ and I′-N′, represent cells containing activated caspases. Blue, DAPI staining. (A-G) Fluorescence of LG. (A-C,A′-C′) Control LG expressing *Drs* (*r4>Drs*) (A,A′), Def (*r4>Def*) (B,B′) and *Dpt* (*r4>Dpt*) (C,C′). Notably, no apoptotic signals are evident. LGs of *mxc^mbn1^* larvae expressing GFP (D,D′), *Drs* (E,E′), *Def* (F,F′) and *Dpt* (G,G′) in their fat bodies. The LG of *mxc^mbn1^* contains many apoptotic signals (B,B′). LGs of *mxc^mbn1^* larvae overexpressing *Drs* (C,C′), *Def* (D,D′) and *Dpt* (E,E′) show more apoptotic signals than *mxc^mbn1^* larvae. (H) Proportion of apoptotic area in total LG area (Welch's *t*-test, **P<0.01, ***P<0.001). The error bars represent s.e.m. Red horizontal lines represent the mean. (I-K) Fluorescence of wing and haltere discs. (L-N) Fluorescence of eye discs. No apoptotic signals can be seen in the wing and haltere discs or eye discs in the control larvae overexpressing *Drs* or *Dpt* genes in their fat bodies. Scale bars: 100 μm.

To test the hypothesis that AMPs encoded by target genes of innate immune pathways are responsible for the suppressive effect on the development of the LG tumours in *mxc^mbn1^* larvae, we examined whether the AMPs could stimulate apoptosis in the tumour cells. LG cells containing activated caspases in mature larvae were detected using the Cell Event Caspase-3/7 Detection assay. No apoptotic signals were seen in normal LGs in the control larvae expressing either the *Drs*, *Def* or *Dpt* gene in the fat body (*n*>20 for each) (Fig. 6A-C,A′-C′). By contrast, we observed apoptotic signals in 7.7% of the whole LG region in *mxc^mbn1^* larvae expressing GFP in fat bodies at the wandering stage of 3rd instar (*n*=20) (Fig. 6D,D′,H). Interestingly, in *mxc^mbn1^* larvae that were at the same larval stage and overexpressing *Drs*, *Def* and *Dpt* specifically in fat bodies, an increase in apoptotic signals was observed over a wider region of the whole LG, as compared with *mxc^mbn1^* without induced AMP expression [14.8%, *n*=20 (*mxc^mbn1^/Y; r4>Drs*); 17.8%, *n*=18 (*mxc^mbn1^/Y; r4>Def*); 16.3%, *n*=22 (*mxc^mbn1^/Y; r4>Dpt*)] (Fig. 6E-G,E′-G′,H). However, we did not observe any apoptotic signals in the control larvae overexpressing *Drs* or *Dpt* in their fat bodies (*n*>9) (Fig. 6A). Despite overexpression of each of the AMPs, the larval mortality could not be rescued in *mxc^mbn1^* larvae (*n*<100 larvae), as it had no effects on the reduced tissue growth in their imaginal discs. Using anti-cleaved caspase 3 immunostaining, we further confirmed these results that overexpression of *Def* in the fat bodies substantially enhanced the induction of apoptosis in the LGs of *mxc^mbn1^*, but not in LGs or imaginal discs of *mxc^+^* controls (Figs S4 and S5).

**Fig. 7.**
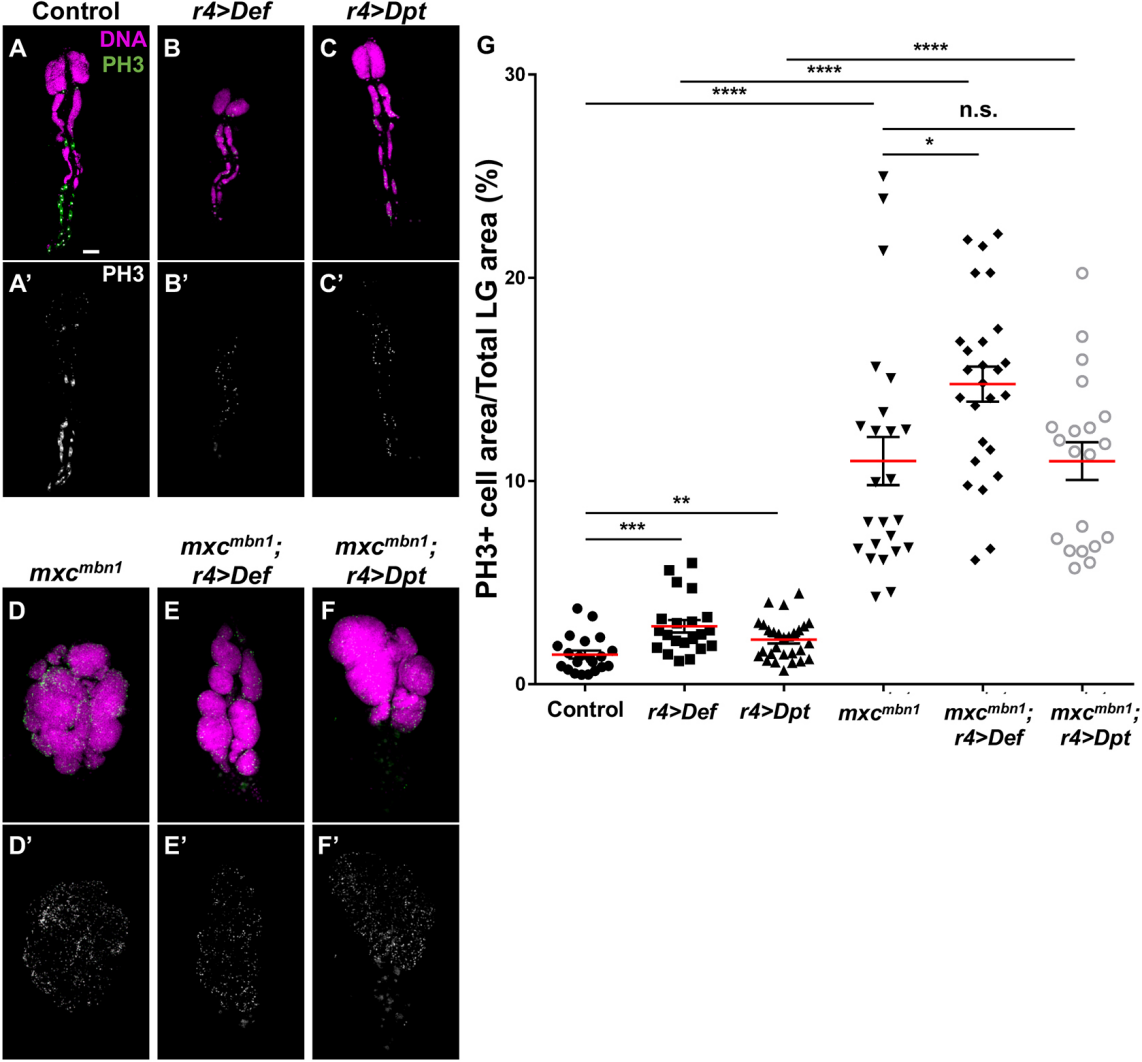
**Anti-PH3 immunostaining of LGs from mature larvae to visualise mitotic cells.** (A-C′) LGs from 3rd instar larvae at a mature stage. A whole LG from normal control larva (A), whole LG lobes from *r4>Def* (B) and whole LG lobes from *r4>Dpt* (C). (D-F) Anterior lobes of LGs from *mxc^mbn1^* larvae (D), *mxc^mbn1^* overexpressing *Def* (E) and *mxc^mbn1^* overexpressing *Dpt* (F) at mature 3rd instar stage. Magenta, DAPI staining; green, anti-PH3 (Ser10) immunostaining. (G) Percentage of the area occupied by PH3-positive cells in LG lobes within the total area of LG lobes (except pericardial cells). The error bars represent s.e.m. Red horizontal lines represent the mean. (Welch's *t*-test; n.s., not significant, *P<0.05, **P<0.01, ***P<0.001, ****P<0.0001). Scale bar: 100 μm.

Moreover, to exclude the possibility that overexpression of AMPs in the fat bodies suppressed cell proliferation in the LGs of the mutant, we examined whether the number of mitotic cells decreased after the ectopic expression of either *Def* or *Dpt* in the fat bodies (Fig. 7). Consequently, the ectopic expression of *Dpt* in the mutant larvae did not lead to a significant alteration in the number of phosphohistone H3 (PH3)-positive cells (Fig. 7D,F,G). In the LG of *mxc^mbn1^* overexpressing *Def* in fat bodies, the number of PH3-positive cells increased compared with those in *mxc^mbn1^* (*P*<0.05, Welch's *t*-test) (Fig. 7D,E,G). From these results, we conclude that AMPs have a cytotoxic effect and can induce apoptosis exclusively in tumour cells.

### Preferential association of AMPs with regions showing a reduced cell density and possessing lower levels of DE-cadherin in the LG tumours

**Fig. 8.**
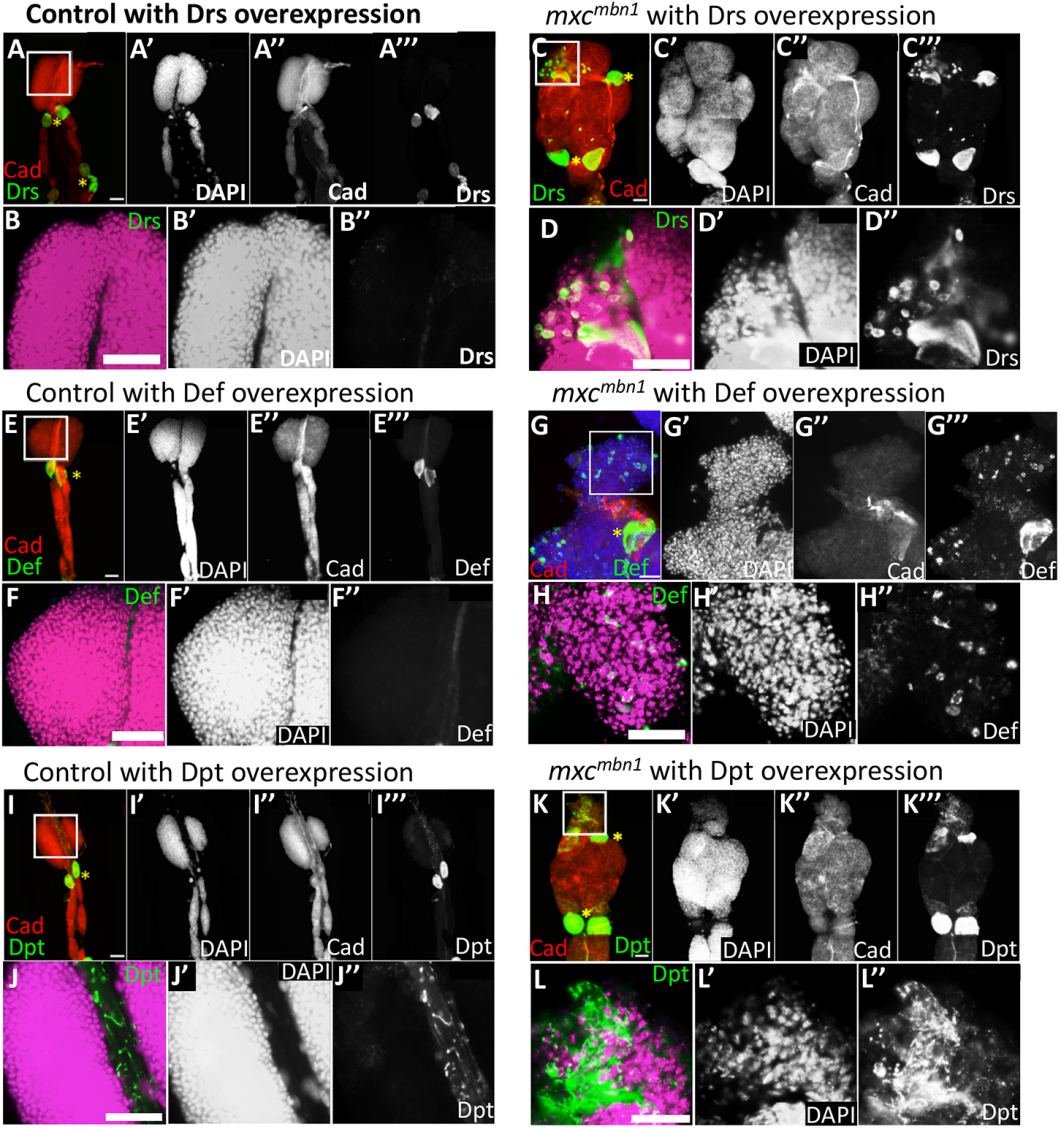
**Visualisation of AMP on LGs and in circulating haemocytes from normal control and *mxc^mbn1^* larvae overexpressing AMPs.** (A-L″) Localisation on LGs of AMPs fused with HA-tag showing control LG (*r4>Drs*) (A,B), LGs from *mxc^mbn1^* overexpressing *Drs* (*mxc^mbn1^/Y; r4>Drs*) (C,D), control LG (*r4>Def*) (E,F), LGs from *mxc^mbn1^* overexpressing *Def* (*mxc^mbn1^/Y; r4>Def*) (G,H), control LG (*r4>Dpt*) (I,J) and LGs from *mxc^mbn1^* overexpressing *Dpt* (*mxc^mbn1^/Y; r4>Dpt*) (K,L). (A,C,E,G,I,K) Red, DE-cadherin (Cad); green, AMP (Drosomycin, Defensin or Diptericin); blue (G), DAPI staining. (B,D,F,H,J,L) High-magnification views (of boxed areas in A,C,E,G,I,K, respectively), showing the DNA (magenta) and AMP (green) in the pericardial cells. AMPs are often localised on regions exhibiting reduced DE-cadherin immunostaining signals. Drosomycin and Defensin appear to be taken into haemocyte-like cells (D,H), whereas Diptericin is associated with the LG in a fibrous shape (H). Scale bars: 50 μm.

As the AMPs secreted into the haemolymph can induce apoptosis in the LG tumour cells but not in normal LG cells, we addressed the mechanism associated with the specific induction of apoptosis in the tumour LGs. AMPs are mainly synthesised in the fat-body cells and secreted into the haemolymph; therefore, these AMPs act on the LG tumours via the haemolymph in *mxc^mbn1^* larvae. We induced the expression of Drosomycin, Defensin and Diptericin fused with a haemagglutinin (HA)-tag in the fat bodies of the control *mxc^mbn1^* mutant larvae (*r4>AMP-HA* and *mxc^mbn1^/Y; r4>AMP-HA*). These tagged AMPs, which were produced in and secreted from the fat bodies, were detected using anti-HA antibody immunostaining. We failed to find any signals in the control LGs (*r4>Drs-HA*, *r4>Def*-HA, *r4>Dpt-HA*) (Fig. 8A,B,E,F,I,J) except in pericardial cells, which have a role in pumping haemolymph into the open circulatory system (asterisks in Fig. 8A,C,E,G,I,K). The presence of Drosomycin in the pericardial cells suggests that these AMPs in haemolymph are preferentially incorporated into the pericardial cells. By contrast, we saw distinct immunostaining signals of the three AMPs in the LG tumours in *mxc^mbn1^* larvae overexpressing the AMPs with a HA-tag (Fig. 8C,G,K). In particular, we observed the AMP signals along the LG regions in the mutant, which showed reduced cell density (Fig. 8C′,G′,K′) and a reduced DE-cadherin distribution in *mxc^mbn1^* (Fig. 8C″,G″,K″). Upon close observation of the LG samples under the microscope at high magnification, we found that Drosomycin and Defensin were taken up by haemocyte-like cells associated with the LG regions in *mxc^mbn1^* (Fig. 8D,H). Many small punctate signals corresponding to these two AMPs were also observed in the LGs. Diptericin was similarly associated with the LG, forming fibrous short fragments (Fig. 8L). Even in the controls overexpressing Diptericin, similar short fragments as well as many small dots containing the AMP were found in dorsal vessels, but not in the LGs (Fig. 8J).

**Fig. 9.**
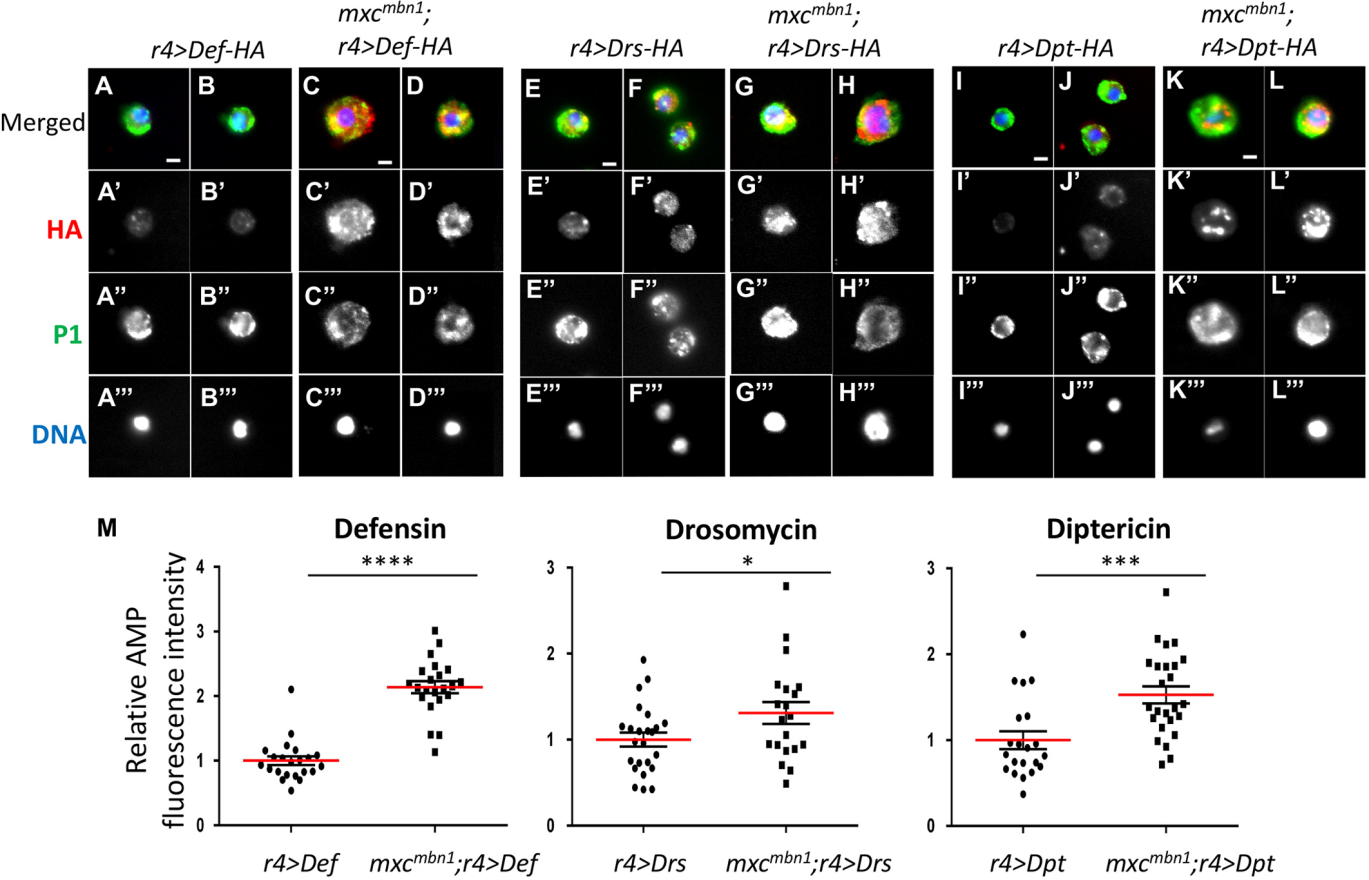
**Accumulation of AMPs in circulating haemocytes from normal control and *mxc^mbn1^* larvae overexpressing AMPs in their fat bodies.** (A-L‴) The cellular localisation of AMPs fused with a HA-tag in circulating haemocytes immunostained with anti-P1 antibody. Two typical examples of the haemocytes are shown for each genotype. (A-D) Circulating haemocytes from larvae overexpressing HA-tagged Def in their fat bodies, control (*r4>Def*) (A,B) and *mxc^mbn1^/Y; r4>Def* (C,D). (E-H) Circulating haemocytes from larvae overexpressing HA-tagged Drs in their fat bodies, control larvae (*r4>Drs*) (E,F) and *mxc^mbn1^/Y; r4>D*rs (G,H). (I-L) Circulating haemocytes from larvae overexpressing HA-tagged Dpt in their fat bodies, control (*r4>Dpt*) (I,J) and *mxc^mbn1^/Y; r4>Dpt* (K,L). Red, anti-HA immunostaining to recognise each AMP; green, anti-P1 immunostaining to recognise circulation plasmatocytes; blue, DAPI staining. (M) Quantification of the fluorescence intensity of anti-HA immunostaining images from larvae overexpressing Defensin, Drosomycin and Diptericin. Student's *t*-test, *P<0.05, ***P<0.001, ****P<0.0001. The error bars represent s.e.m. The horizontal red lines represent the mean. Scale bars: 10 μm.

After finding the haemocyte-like cells containing either Drosomycin or Defensin on the LG tumours in *mxc^mbn1^* larvae, we examined whether circulating haemocytes in the haemolymph also contained AMPs. We induced Drosomycin, Defensin and Diptericin with an HA-tag exclusively in larval fat bodies and examined their accumulation in circulating haemocytes by using anti-HA-tag immunostaining. We visualised the haemocytes by simultaneous immunostaining with anti-P1 antibody, which is a plasmatocyte-specific marker. The intensity of the anti-HA immunostaining signal of Defensin that appeared in the cytoplasm of circulating haemocytes in the tumourous *mxc* mutant (*mxc^mbn1^/Y; r4>Def-HA*) was higher than in controls (*r4>Def-HA*) (Fig. 9A-D,M, *P*<0.0001, *n*>20). A less remarkable but significantly higher signal of Drosomycin appeared in haemocytes from *mxc^mbn1^* larvae (*mxc^mbn1^/Y; r4>Drs*) than in controls (*r4>Drs*) (*P*<0.05, *n*>20) (Fig. 9E-H,M). In addition, a significantly higher signal of Diptericin was also seen in haemocytes from the mutant larvae (*mxc^mbn1^/Y; r4>Dpt*) (Fig. 9I-L,M), although we did not observe the haemocytes containing this AMP in the LGs. To confirm that these HA-tagged AMPs were induced at a similar level by *r4-Gal4* in the controls and mutants, we performed qRT-PCR. The level of each mRNA increased remarkably in both *r4>AMP* and *mxc^mbn1^/Y; r4>AMP*, compared with levels in control larvae (Fig. S6). The differences between the mRNA levels in *mxc^+^* and *mxc^mbn1^* were not statistically significant in the case of each AMP. Nevertheless, the haemocytes in the *mxc^mbn1^* larvae contained significantly higher amounts of AMPs (Fig. 9M). Taken together, these results indicate that the AMPs produced in the fat body are secreted into the haemolymph of the larvae. Subsequently, the AMPs in haemolymph can be incorporated in a preferential manner into circulating haemocytes in the *mxc^mbn1^* larvae with the LG tumour. The haemocytes were associated with the LG regions showing reduced cell density.

## DISCUSSION

### Innate immune systems can suppress the development of tumours generated in a larval haematopoietic tissue in *Drosophila*

Our molecular data showed the expression of six target genes corresponding to two major innate immune signalling pathways, which arise simultaneously in the *mxc^mbn1^* mutants, indicating that the innate immune pathways acting upstream of the AMP genes were activated in the LG tumour mutants. This activation was required for suppression of tumour growth. The ectopic expression of the AMPs in fat bodies significantly suppressed the tumour phenotype, as expected. This genetic data led us to hypothesise that there exists a regulatory mechanism by which the innate immune pathways are activated and induce the expression of the AMP genes in the presence of tumours. Thus, it seems reasonable to speculate that the innate immune systems have anti-tumour effects that can suppress the hyperproliferation of malignant cells in *Drosophila*. Our genetic data imply that a defence mechanism against the tumour cells exists even in insects, despite the lack of an acquired immune system. If the innate immune system in insects has a role as a primitive prophylactic measure against tumour cells, it would be advantageous to these organisms which lack the more advanced immune systems acquired by vertebrates during the course of evolution (Hoffmann and Reichhart, 2002). As tumourigenesis is an inevitable feature for multicellular organisms (Domazet-Lošo et al., 2014), it is crucial to suppress the uncontrolled proliferation of abnormal cells that deviate from their proper role in a multicellular system. Nevertheless, few biological studies have addressed the means by which invertebrates challenge tumour cells. Interestingly, our findings raise the possibility that *Drosophila* has a biological defence system that could recognise tumour cells. Several studies have provided evidence supporting this possibility. Haemocytes were found to adhere to the tumours generated in the imaginal discs upon disruption of the basement membrane, and subsequently their growth was restricted (Parisi et al., 2014; Pastor-Pareja et al., 2008). In solid epithelial tumours, the Toll-signalling pathway was seen to be activated in fat-body cells causing cell death of these tumours and a reduction in their size (Parisi et al., 2014). Another study reported that haemocytes were recruited by tumour-like tissues expressing oncogenic Ras^V12^ and this led to an encapsulation reaction, a cellular response usually observed after infection with large foreign objects (Hauling et al., 2014). These studies show the mechanisms by which *Drosophila* can recognise tumour cells in the body, which is an important subject. The innate immune system might recognise more common disorders, such as the loss of tissue integrity as seen in the case of breaks in the epidermal barrier or stress-related signal(s) induced by cell damage (Capilla et al., 2017). If this is the case, we might be able to clarify why the two independent innate immune pathways that are intrinsically triggered by different types of microorganisms were simultaneously activated in the *mxc^mbn1^* mutant larvae. It is fascinating to explore the mechanisms by which the innate immune system can recognise the tumour cells in *Drosophila*.

### Drosomycin and Defensin secreted from fat bodies into the haemolymph act on the tumours via circulating haemocytes

The ectopic expression of each of the five AMPs in fat bodies of the *mxc^mbn1^* mutants significantly suppressed the LG tumour phenotype, and also enhanced apoptosis specifically in the tumourous LG of *mxc^mbn1^*. Interestingly, haemocyte-like cells containing Drosomycin and Defensin were associated with the LG tumours. Larger amounts of AMPs were also found in the cytoplasm of circulating haemocytes in the mutants, although AMPs synthesised in the fat body are secreted into the haemolymph (Lemaitre and Hoffmann, 2007). These observations suggest that Drosomycin and Defensin are preferentially taken into the circulating haemocytes in the mutant larvae. This finding that AMPs accumulate in the cytoplasm of circulating haemocytes is surprising, as such accumulation can induce disruption of bacterial membranes. Subsequently, the haemocytes were recruited into the LG tumours. Previous studies have reported that *Drosophila* AMPs stored in the cytoplasmic granules of circulating haemocytes were discharged against infectious microbes (Destoumieux et al., 2000; Yakovlev et al., 2017). Moreover, AMP-containing haemocytes are reportedly recruited to neoplastic tumours in *Drosophila* (Cordero et al., 2010; Parisi et al., 2014; Pastor-Pareja et al., 2008). The haemocytes release the stored AMPs against the tumour cells by regulated exocytosis (Destoumieux et al., 2000; Yakovlev et al., 2017). In the present study, we showed that Drosomycin and Defensin have an anti-tumour effect against the haematopoietic tumours with the help of circulating haemocytes in the haemolymph. In our study, however, it is difficult to investigate interactions between the haemocytes and the cells of the tumourous tissues, as both cell types share a common developmental origin. In addition, in the *mxc^mbn1^* mutant some of the circulating haemocytes are abnormally differentiated and display the malignant phenotype (Remillieux-Leschelle et al., 2002). This differentiation complicates analysis of the anti-tumour effect of AMPs through haemocytes. Therefore, it will be important to examine the effects of AMPs against tumours generated in different tissues, such as imaginal discs in larvae, which have normal haemocytes.

### How does Diptericin specifically target tumour cells?

By contrast to the mechanism of action of Drosomycin and Defensin against the LG tumours, Diptericin seems to exert its anti-tumour effect by a different mechanism. It appears to act more directly on the tumours without the intervention of circulating haemocytes. Nevertheless, the effect of Diptericin is restricted to the tumour cells and does not extend to normal cells. Despite the fact that the AMPs are incorporated into circulating haemocytes, it is still unclear why the haemocytes containing the AMP were not associated with the LG tumours. According to a proposed hypothesis, the AMPs have a higher affinity for the tumour cells than for normal cells because of a difference in the electric charge on the plasma membrane (Rivero-Müller et al., 2007). Indeed, it was seen that some tumour cells have a net negative charge on the plasma membrane, similar to bacterial cells (Hoskin and Ramamoorthy, 2008; Mader and Hoskin, 2006; Papo and Shai, 2005). We carried out staining experiments using the fluorescent dye PSVue550, which is a probe known to bind to several negatively charged phospholipids (www.mtarget.com/mtti/documents/PSVue_extended_May22012_000.pdf). However, we failed to detect any difference in the staining intensity between the tumour LGs from *mxc^mbn1^* and normal LGs (data not shown). Therefore, it will be necessary to explore the possibility of a new mechanism for the restricted association of Diptericin with LG tumours in our future studies. We hope to elucidate the mechanism underlying the association between AMP overexpression and apoptosis induction in our further studies.

### AMPs as promising anticancer drugs

As a variety of AMPs are found in many organisms from plants to humans, they are regarded as common protective factors that are essential for the initial defence against foreign cells by the innate immune system (Hoskin and Ramamoorthy, 2008; Mader and Hoskin, 2006). In the present study, we provided evidence that AMPs are induced as a consequence of activation of the innate immune pathways in response to haematopoietic tumours in *Drosophila*. Our observations showed that three different AMPs enhanced apoptosis in the tumours through two different routes, either with the aid of circulating haemocytes or through direct binding to the tumour cells. Although the mechanism of action of mammalian AMPs has not yet been elucidated, they have been shown to be involved in the immune response not only against microbial pathogens but also against cancer cells (Winder et al., 1998; Zaiou, 2007). Some human AMPs also display anticancer activity that induces cell death in cultured cancer cells (Baker et al., 1993; Sloballe et al., 1995). Other AMPs have concentration-dependent cytotoxic effects on mammalian cancer cells at concentrations lower than that required for normal cultured cells (Hoskin and Ramamoorthy, 2008; Mader and Hoskin, 2006). Although the acquired immune system in mammals is more effective for preventing cancer progression, it is also possible that the innate immune system makes a greater contribution than previously assumed. In addition, one of the most serious problems in cancer chemotherapy is the strong side effects of anticancer drugs. In this context, our data indicate that overexpression of the three AMPs had no adverse effects on the normal *Drosophila* tissues. We demonstrated that Drosomycin and Defensin act on tumour cells through circulating haemocytes. It is likely that these macrophage-like cells can be specifically associated with tumours and that the AMPs accumulated in the cells are discharged into the tumours. We also demonstrated that Diptericin acts directly on tumour cells in *Drosophila*. Considering past and present results, AMPs are attractive therapeutic candidates for cancer treatment (Gaspar et al., 2013). As most biological studies on AMPs have been performed using cultured cell lines, however, it will be essential to obtain data on AMPs at the organism level. The experimental system established in this study allows us to investigate the anticancer effects of AMPs in *Drosophila* and therefore provides a fascinating model for future cancer research.

### Conclusion

To our knowledge, this is the first study to present genetic evidence to suggest that the innate immune systems are activated in response to haematopoietic tumours in *Drosophila*. This activation is essential for the suppression of tumour progression. Moreover, the anti-tumour effect is exerted through the induction of AMPs, which are components of two major innate immune pathways. The AMPs secreted into the haemolymph are preferentially associated with LG tumours by direct binding or with the aid of circulating haemocytes. Ectopic overexpression of the AMPs enhanced apoptosis of cells in the LG of *mxc^mbn1^*, but not in normal tissues. On the basis of these results, we propose that in *Drosophila* there is a surveillance system that activates the innate immune system in response to tumour cells. These AMPs could represent promising new anti-cancer agents that do not have side effects.

## MATERIALS AND METHODS

### Drosophila stocks

All *D. melanogaster* stocks were maintained on standard cornmeal food, as previously described (Oka et al., 2015). Canton S was used as the wild-type stock; *w^1^* was used as the normal control stock. A recessive lethal allele of *mxc* showing the LG tumour phenotype (*mxc^mbn1^*) was obtained from *w^1^ mxc^mbn1^; Dp(1;4)mxc^+R^* stock (#108753, Kyoto Stock Centre, Kyoto, Japan) after removing the duplication. A hypomorphic allele (*mxc^G43^*), which did not display any tumour phenotype, was obtained from the Bloomington *Drosophila* Stock Centre (BDSC) (#32183, Bloomington, IN, USA). To induce expression of the AMP genes *Drs*, *Def* and *Dpt* using the Gal4/UAS system, three UAS stocks were employed: *M{UAS-Drs.ORF.3xHA.GW}* (#F002219), *M{UAS-Def.ORF.3xHA.GW}* (#F002467) and *M{UAS-Dpt.ORF.3xHA.GW}* (#F002330) from the FlyORF (Zurich, Switzerland). Other UAS-AMP stocks for the induction of Metchnikowin and Attacin A were gifts from Dr B. Lemaitre (Swiss Federal Institute of Technology). For the genetic analysis, we used four mutants, *dl^4^* (#BL7096; BDSC), *Tl^r3^* (#BL3238; BDSC), *Tl^rv1^* (a gift from Dr Y. Yagi, Nagoya University) and *imd^1^* (a gift from Dr Y. Yagi), to modify activity of the innate immunity pathways. For overexpression of innate immunity genes, we used *UAS-Dl* (#F000638; FlyORF), *UAS-Tl^10B^* (#BL58987; BDSC) and *UAS-Rel.68* (#BL55778; BDSC). Three Gal4 driver stocks were used for ectopic expression in specific larval tissues: *ppl-Gal4* for expression in the fat body at a higher level (a gift from Dr P. Léopold, Institute Valrose Biologie), *P{w[+mC]=r4-GAL4}3* (*r4-Gal4*) for fat-body-specific expression at a moderate level (#BL33832; BDSC) and *P{Lsp2-GAL4}3* (*Lsp2-Gal4*) for fat-body-specific expression in the 3rd instar larvae at a weaker level (#BL6357; BDSC). *Hml-Gal4* was used for expression restricted to the cortical zone of the 1st lobe and *upd3-Gal4* was used for expression in the medullary zone of the lobe (a gift from Dr D. Hultmark, Umea University). To monitor the activation of the innate immune pathways, two reporters for AMP genes were employed: *Drosomycin-YFP* (*Drs-YFP*) (a gift from Dr Y. Yagi) and *Diptericin-YFP* (*Dpt-YFP*) (a gift from Dr Y. Yagi).

### LG preparation

Normal controls (*w/Y*) pupated at 6 days (28°C) and 7 days (25°C) after egg laying (AEL), whereas some of the *mxc^mbn1^* mutant remained in 3rd instar larval stage at 8 days (28°C) and 10 days (25°C) AEL. To minimise the possibility of a delay that might allow the hyperplastic tissue to grow, the comparative analysis of hemizygous mutants and controls was performed on the same day, when the wandering 3rd instar larval stage was seen. Alternatively, the tissues were collected from hemizygous mutant larvae 1 day after the timing of the LG collection from control larvae. For the staging of the larvae, parent flies were transferred into a new culture vial and left there to lay eggs for 24 h. Careful attention was given to avoid overcrowding of the larvae in the vial. To compare the size of LGs, a pair of anterior lobes of the LG without connected cardiac cells from mature stage larvae were isolated and fixed with 3.7% formaldehyde for 5 min. The fixed samples were mildly flattened under constant pressure using an apparatus so that the tissue became spread out into cell layers with a constant thickness.

### RNA-seq analysis

Total RNA was extracted from the 3rd instar larvae using the Trizol^®^ reagent (Invitrogen). The isolated RNAs were used for the construction of single-end mRNA sequence libraries, using a NEBNext Ultra Directional RNA Library Prep Kit (New England Biolabs) according to the manufacturer's recommendations. Sequence analysis of the mRNA was performed on an Illumina Hi-seq 1000 instrument using 51 bp single-end reads. The read quality was checked for each sample using FASTQC (http://www.bioinformatics.babraham.ac.uk/projects/fastqc) (version 0.11.5). Filtered reads were aligned to the reference *D. melanogaster* genome sequence (BDGP5) using the TopHat program version 2.0.6 with default parameters (Kim et al., 2013). Cufflinks (version 2.0.2) was employed with default parameters for transcript assembly (Trapnell et al., 2012). The expression level of each gene was quantified as FPKMs (fragments per kilo base of exon per million mapped fragments). We carried out gene ontology (GO) analysis of genes expressed in the adults and aligned the sequences for the genes with significant changes in expression (*q*-values <0.01). The GO classification system was applied employing the database for annotation, visualisation and integrated discovery (DAVID) version 6.7 (http://david.abcc.ncifcrf.gov/).

### qRT-PCR analysis

Total RNA was extracted from the 3rd instar larvae of each genotype using the Trizol^®^ reagent (Invitrogen). Synthesis of cDNA from the total RNA was carried out using the PrimeScript^TM^ High Fidelity RT-PCR Kit (Takara Bio) and oligo dT primers. RT-PCR was performed using the FastStart Essential DNA Green Master (Roche) and a Light Cycler Nano instrument (Roche). The qRT-PCR primers were synthesised as follows: RP49-Fw, 5′-TTCCTGGTGCACAACGTG-3′; RP49-Rv, 5′-TCTCCTTGCGCTTCTTGG-3′; Drosomycin-Fw, 5′-GTACTTGTTCGCCCTCTTCG-3′; Drosomycin-Rv, 5′-CAGGGACCCTTGTATCTTCC-3′; Defensin-Fw, 5′-CTTCGTTCTCGTGGCTATCG-3′; Defensin-Rv, 5′-CCAGGACATGATCCTCTGGA-3′; Diptericin-Fw, 5′-CAGTCCAGGGTCACCAGAAG-3′; Diptericin-Rv, 5′-AGGTGCTTCCCACTTTCCAG-3′; Metchnikowin-Fw, 5′-TACATCAGTGCTGGCAGAGC-3′; Metchnikowin-Rv, 5′-ACCCGGTCTTGGTTGGTTAG-3′; AttacinA-Fw, 5′-ACTACCTTGGATCTCACGGGA-3′; AttacinA-Rv, 5′-TGATGAGATAGACCCAGGCCA-3′; Cecropin-Fw, 5′-ATCGGAAGCTGGTTGGCTAAA-3′; Cecropin-Rv, 5′-CTGGGTACTCCATCGACCATG-3′. Each sample was duplicated on the PCR plate and the final results average three biological replicates. For the quantification, the ΔΔCt method was used to determine the difference between target gene expression relative to the reference *Rp49* gene expression.

### LG immunostaining

For immunostaining of the larval LGs, a pair of anterior lobes of the LGs were dissected from matured 3rd instar larvae and fixed in 4% paraformaldehyde in phosphate-buffered saline (PBS) for 15 min at 25°C. After repeated washing, samples were blocked with PBS containing 0.1% Triton X-100 and 10% normal goat serum and the fixed samples were incubated with primary antibodies at 4°C overnight. Four primary antibodies were employed: anti-DE-cadherin monoclonal antibody (1:300; Developmental Studies Hybridoma Bank, Cat#DCAD2-c), anti-phospho-Histone H3 (Ser10) antibody (1:1000; Merk-Millipore, 06-570), anti-HA-tag rabbit IgG (1:200; Cell Signaling Technology, 3724) and anti-cleaved caspase 3 antibody (1:200; Cell Signaling Technology, 9661). After extensive washing, specimens were incubated with Alexa 594 or Alexa 488 secondary antibodies (1:400; Molecular Probe, A-32754 and A-11029, respectively). The LG specimens were placed on a fluorescence microscope (Olympus, model IX81), fitted with excitation and emission filter wheels (Olympus). The fluorescence signals were collected using a 10×, 20×, 40× or 60× dry objective lens. Specimens were illuminated with UV-filtered and shuttered light using the appropriate filter wheel combinations through a GFP/RFP filter cube. Near simultaneous GFP and/or RFP fluorescence images were captured with a CCD camera (Hamamatsu Photonics). Image acquisition was controlled through the Metamorph software version 7.6 (Molecular Devices) and processed with ImageJ or Adobe Photoshop CS.

### Immunostaining of circulating haemocytes

After washing the 3rd instar larvae with PBS, a single larva was transferred into the *Drosophila* Ringer solution (DR) [10 mM Tris-HCl (pH 7.2), 3 mM CaCl_2_ 2H_2_O, 182 mM KCl, 46 mM NaCl] on a glass slide. Subsequently, only the larval epidermis was cut by a set of fine forceps so as to allow the circulating haemocytes to be released into the DR outside the larvae. After an aliquot of the DR containing circulating haemocytes was placed on the glass slide, it was evaporated by exposure to hot air and the haemocytes were fixed in 4% paraformaldehyde for 5 min at 25°C. Immunostaining of the haemocytes was carried out using both anti-P1 monoclonal antibody (Kurucz et al., 2007; 1:100) and anti-HA-tag rabbit IgG (1:200) as described above.

### Apoptosis assay

Cells undergoing apoptosis were detected using a Cell Event Caspase-3/7 Green Detection Reagent (Molecular Probes, Invitrogen). The 3rd instar larvae were dissected in PBS to collect LGs or imaginal discs. The tissues were then incubated in PBS containing 2 μM Cell Event Caspase-3/7 Green Detection Reagent for 30 min at 37°C. After incubation, the LGs or imaginal discs were fixed with 4% paraformaldehyde for 15 min at 25°C. Following fixation, the tissue specimens were repeatedly washed and permeabilised in PBST (PBS containing Tween). After several washings, the specimens were mounted and observed as described above. Immunostaining of LGs with anti-cleaved caspase 3 antibody was carried out as described previously.

### Statistical analysis

For the LG area measurements, more than 20 larvae were used in each genotype. Results were presented as bar graphs or scatter plots created using GraphPad Prism 6. The area in pixels was calculated and an average determined for each LG. Each single dataset was assessed using Welch's *t*-test or Student's *t*-test. An *F*-test was performed to determine equal or unequal variance. If the *F*-value was greater than 0.05, then *P*-values were calculated using the Student's *t*-test of equal variance. If the value was less than 0.05, then *P*-values were calculated using Welch's *t*-test of unequal variance. Statistical significance is described in each figure legend as follows: *P<0.05, **P<0.01, ***P<0.001 and ****P<0.0001.
